# Sleep quality is associated with reduced quality of life in inflammatory bowel disease through its interaction with pain

**DOI:** 10.1002/jgh3.70021

**Published:** 2024-08-24

**Authors:** Alex Barnes, Robert V Bryant, Sutapa Mukherjee, Paul Spizzo, Réme Mountifield

**Affiliations:** ^1^ Department of Gastroenterology Southern Adelaide Local Health Network (SALHN) Flinders Medical Centre Bedford Park South Australia Australia; ^2^ College of Medicine and Public Health Flinders University Bedford Park South Australia Australia; ^3^ School of Medicine, Faculty of Health and Medical Sciences University of Adelaide Adelaide South Australia Australia; ^4^ Department of Gastroenterology Queen Elizabeth Hospital Woodville South Australia Australia; ^5^ Adelaide Institute for Sleep Health, Flinders Health and Medical Research Institute College of Medicine and Public Health, Flinders University Bedford Park South Australia Australia; ^6^ Department of Respiratory and Sleep Medicine Southern Adelaide Local Health Network (SALHN) Flinders Medical Centre Bedford Park South Australia Australia

**Keywords:** inflammatory bowel disease, insomnia, pain interference, quality of life

## Abstract

**Background and Aim:**

Quality of life is reduced in people with inflammatory bowel disease (IBD) and poor sleep is prevalent in people with IBD. This study aimed to investigate the influence of sleep on quality of life (QoL) in people with inflammatory bowel disease.

**Methods:**

An online questionnaire was administered through three tertiary IBD centers, social media, and through Crohn's Colitis Australia. The questionnaire included the EQ‐5D‐5L measures of health‐related QoL, the Insomnia Severity Index, the Pittsburgh Sleep Quality Index (PSQI), and validated IBD activity and mental health scores.

**Results:**

There were 553 responses included with a diagnosis of Crohn's disease (62.2%), with over half on biologic therapy (53.1%). Poor sleep and clinically significant insomnia were associated with lower QoL (EQ‐5D‐5L scores: EQVAS, utility score, *P* < 0.001 for all). Sleep quality scores correlated with the EQ‐5D‐5L domains of “pain” (*ρ* 0.35, *P* < 0.001), “usual activities” (*ρ* 0.32, *P* < 0.001), and “depression‐anxiety” (*ρ* 0.37, *P* < 0.001). After adjusting for demographic variables, IBD activity, and depression and anxiety via multivariate regression, the “pain” domain continued to be associated with PSQI components “sleep quality” (*P* < 0.001), “sleep disturbance” (*P* < 0.001), and “sleep duration” (*P* < 0.001). Clinically significant insomnia was associated with a reduction in QoL (EQVAS, utility score) independent of IBD activity (*P* < 0.001) and of a similar magnitude to that seen with IBD activity.

**Conclusion:**

Health‐related QoL in IBD is influenced by aspects of sleep quality irrespective of IBD activity and mental health conditions. The presence of insomnia is associated with a reduction in health‐related QoL. Consideration should be given to sleep targeting interventional studies in an IBD population.

## Graphical abstract

Health‐related quality of life in inflammatory bowel disease (IBD) is influenced by aspects of sleep quality irrespective of IBD activity and mental health conditions. The presence of insomnia is associated with a reduction in health‐related quality of life.

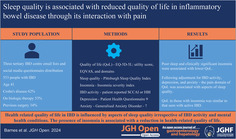

## Introduction

Inflammatory bowel disease (IBD) is an immune‐mediated condition with a chronic relapsing remitting course. The symptoms of active IBD are characterized by diarrhea, abdominal pain, and gastrointestinal bleeding and have a substantial impact on quality of life.^1^, ^2^, ^3^ The impact of IBD disease activity on quality of life (QoL) is expectedly well‐established, with evidence of an improvement in QoL following institution of medical treatment^4^, ^5^, ^6^, ^7^ and surgery.^8^, ^9^, ^10^ However, even in the absence of active disease, people with IBD have a poorer QoL than the general population.^11^, ^12^, ^13^ Factors beyond disease activity are relevant to QoL, in particular, psychosocial dysfunction^14^ and the effect of IBD on relationships with food.^15^

Poor sleep is common in people with IBD^16^ and, while worse in those with clinically active IBD,^17^ it remains an issue for patients in remission.^18^ In people with IBD, disturbed sleep has also been linked to mental health conditions,^19^, ^20^ fatigue,^21^ and opioid usage.^22^ Insomnia is the most common sleep disorder in people with IBD and has been associated with mental health conditions, IBD activity, and worse IBD‐related disability.^23^, ^24^

Poor sleep quality has been associated with decreased health‐related QoL.^26^ A longitudinal study has explored sleep and QoL in IBD, suggesting that sleep apnea and insomnia symptoms were associated with worse health‐related QoL 4 weeks later.^27^ Our study aimed to investigate sleep quality and insomnia severity and its associations with QoL, taking into account IBD activity and mental health conditions.

## Methods

An online questionnaire was made available to people with IBD via tertiary hospital patient email lists, private gastroenterology practice email lists, and social media associated with a patient support organization. Individuals with a self‐reported diagnosis of IBD over 18 years of age were invited to participate. Demographic data such as age and sex were recorded, along with IBD‐related data including disease duration and previous surgery. Ethics approval for this study was obtained from the Southern Adelaide Human Research Ethics Committee (203.20).

The Pittsburgh Sleep Quality Index (PSQI) is a validated tool that assesses perceived sleep quality.^28^ The index consists of subscales on sleep duration, sleep disturbance, sleep latency, daytime dysfunction, sleep efficiency, overall sleep quality, and medications for sleep. The score ranges from 0 to 21, with a PSQI >5 considered to represent poor sleep quality.

The Insomnia Severity Index (ISI) is a self‐reported questionnaire that been validated for assessment of insomnia, evaluating the response to treatment, and as an outcome measure for insomnia research.^29^, ^30^, ^31^ The index consists of seven items with a 5‐point Likert scale used to rate each item. A score between 0 and 7 is considered to indicate the absence of insomnia, 8–14 indicates subthreshold insomnia, 15–21 indicates moderate insomnia, and over 21 denotes severe insomnia. Clinically significant insomnia is defined as an ISI score greater or equal to 10 as is commonly used in screening.^29^

IBD disease activity was assessed using the Harvey–Bradshaw Index (HBI) in the case of Crohn's disease, with HBI > 5 considered active disease.^32^ The patient‐reported version of the HBI was utilized in the survey, although a decision was made to maintain the general well‐being and abdominal pain score similar to the physician HBI rather than using a 10‐point Likert scale.^33^ The Simple Clinical Colitis Activity Index (SCCAI) was used in the case of ulcerative colitis, with an SCCAI >5 considered indicative of active disease.^34^ The patient‐reported form of the SCCAI was utilized^35^ in the survey. The use of a self‐reported SCCAI has been previously validated, with good agreement with physician‐reported SCCAI.^36^

Anxiety was assessed using the Generalized Anxiety Disorder 7‐item scale (GAD‐7),^37^ with a score over 10 used to indicate likely clinically significant anxiety. The Patient Health Questionnaire 9 (PHQ‐9) was used to assess depression, with a score over 15 used to indicate likely clinically significant depression.^38^

QoL was assessed using the EQ‐5D‐5L^39^, which measures health‐related QoL and has been validated in IBD populations,^40^ with mapping available to the short inflammatory bowel disease questionnaire, an IBD‐specific measure of health‐related QoL.^41^ The EQ‐5D‐5L was chosen due to its immediate comparability with other populations and its brevity. The EQ‐5D‐5L consists of five dimensions of health‐related QoL—mobility, usual activities, self‐care, pain, and depression‐anxiety. Each dimension has five levels ranging from no problem (i), slight problems (ii), moderate problems (iii), severe problems (iv), and extreme problems (v). There is also a visual analogue scale of QoL referred to as EQVAS. The EQ‐5D‐5L produces a health state out of 3125 possible health states. The health states are then converted to a single utility index score—here we used the published value set for England.^42^ These health states and utility index scores can then be compared with other populations. Here we used South Australian population norms.^43^

The primary outcome of this study was the relationship between QoL (EQ‐5D‐5L domains) and sleep quality (PSQI components). Secondary outcomes included the relationship between PSQI score and EQ‐5D‐5L scores (utility score and EQVAS), insomnia scores and QoL (EQ‐5D‐5L domains and scores), and examining the influence of IBD activity and depression and anxiety on these relationships.

### Statistical analysis

Statistical analysis was performed using Stata SE 16 (StataCorp, College Station, TX, USA). Inadequate completion of score or index led to that result being discarded. For normally distributed variables, mean and standard deviation (SD) were reported, with comparisons made using the Student's *t*‐test. For non‐normally distributed variables, median and interquartile range (IQR) were reported, with comparisons made using the Mann–Whitney *U* test. Pearson's or Spearman's correlation was used as appropriate, with interpretation of coefficients as follows: very weak <0.19, weak 0.2–0.3, moderate 0.3–0.5, strong 0.5–0.79, and very strong >0.80.^44^ One‐way analysis of variance was used with Tukey post hoc test and adjusted for multiple comparisons as appropriate. Regression was undertaken to determine the predictors of health‐related QoL. Due to multicollinearity, this was performed in a stepwise fashion, with each PSQI component considered separately in univariate regression and then considering other factors such as IBD activity, depression, and anxiety in multivariate regression. Regression was also performed for an outcome of EQVAS and the EQ‐5D utility score to investigate the influence of ISI on these values.

## Results

**Figure 1:**
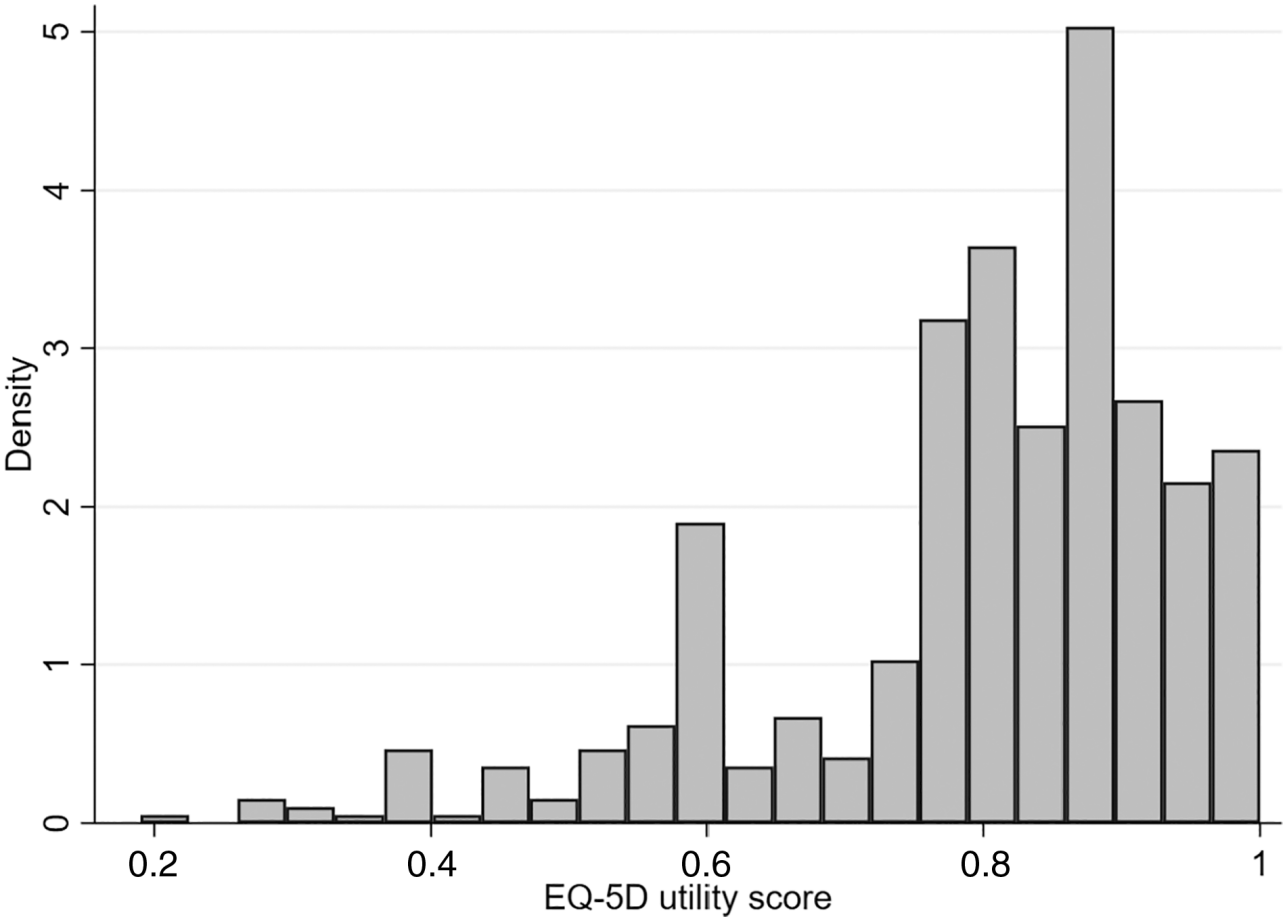
Distribution of EQ‐5D utility scores.

**Figure 2:**
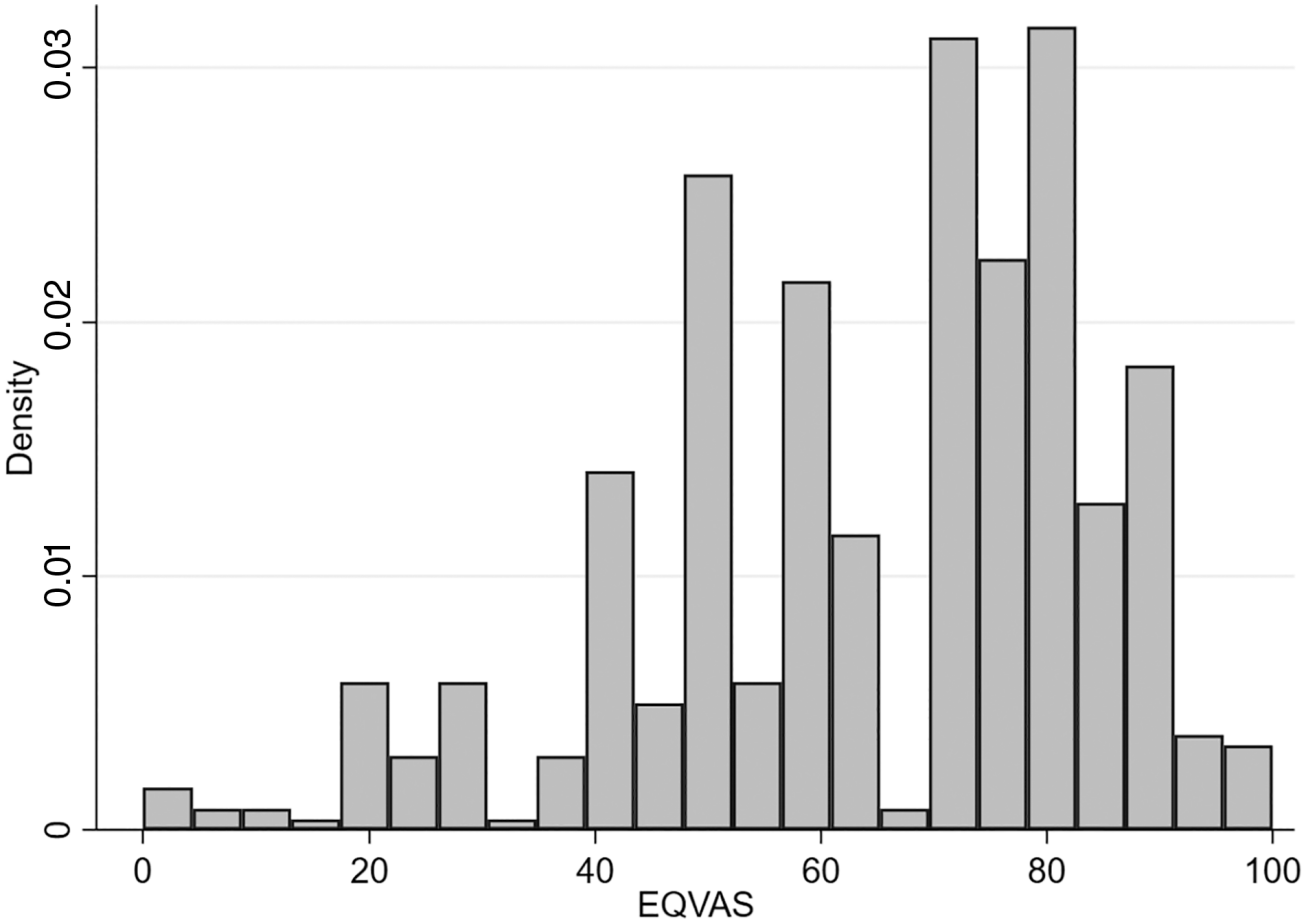
Distribution of EQ‐5D EQVAS score.

The cohort (*n* = 553) was predominantly female (75.4%) with a diagnosis of Crohn's disease (62.2%), with over half on biologic therapy (53.1%) and over a third having had previous surgery for IBD (see Table 1). The completion rate for the questionnaire was 90.5%. Missing or incomplete responses resulted in exclusion of 58 questionnaire responses. EQ‐5D utility score for the cohort was [mean (SD)] 0.79 (0.15) (see Table 2), which was significantly lower than South Australian population norms, with similar results for the EQVAS (64.47 [19.9] *vs* 78.55 [16.57], *P* < 0.001). The distribution of EQ‐5D utility scores and EQVAS can be seen in Figures 1 and 2, and EQ‐5D component scores are given in Figures S1–S4, Supporting information. There was no significant difference in utility scores between Crohn's disease and ulcerative colitis (Table 2), and no significant difference with EQVAS and EQ‐5D component scores, apart from a trend to worse scores for mobility component in Crohn's disease (Table S1).

**Table 1:** Demographics of inflammatory bowel disease population.

IBD and demographic data		Crohn's disease	Ulcerative colitis or indeterminate colitis
Cohort (*n*)	553	343	210
Age [median (IQR)]	41 (32–52)	42 (32–53)	40 (32–50)
Female (%)	75.4	76.3	77.3
Weight [mean (SD)]	78.3 (24.5)	81.1 (20.9)	79.1 (19.8)
Height	162.6 (31.9)	168.2 (9.3)	167.9 (8.8)
Crohn's disease (%)	62.2		
IBD disease duration [mean (SD)]	12.8 (10.5)	14.1 (11.0)*	10.6 (8.9)*
Previous surgery for IBD (%)	34.0	49.5*	9.8*
Biologic usage (%)	53.1	64.3*	37.6*
Immunomodulator usage (%)	33.9	39.0	34.2
Aminosalicyate usage (%)	32.7	11.4*	63.2*
Corticosteroid usage (%)	7.5	7.4	11.5
Alcohol use (%)	35.4	36.7	45.5
Current smoker (%)	7.2	7.7	5.9
Opioid usage (%)	15.2	18.7*	8.9*

* *P* < 0.05.

IBD, inflammatory bowel disease; SD, standard deviation.

**Table 2:** EQ‐5D weighted score [mean (standard deviation)] from South Australia (SA) population norms, entire study cohort, poor sleep quality (Pittsburgh Sleep Quality Index > 5), good sleep quality (Pittsburgh Sleep Quality Index < 5), clinically active inflammatory bowel disease (IBD) (Harvey–Bradshaw Index > 5 or Simple Clinical Colitis Activity Index > 5), clinically inactive IBD (Harvey–Bradshaw Index ≤ 5 or Simple Clinical Colitis Activity Index ≤ 5), clinically significant insomnia (Insomnia Severity Index > 15), and no significant insomnia (Insomnia Severity Index < 7).

	EQ‐5D utility score [mean (standard deviation)]	*P* value
SA population norms	0.91 (0.14)	*P* < 0.001
Entire cohort	0.79 (0.15)	
Crohn's disease	0.80 (0.15)	*P* = 0.35
Ulcerative colitis	0.79 (0.15)	
Poor sleep quality	0.77 (0.15)	*P* < 0.001
Good sleep quality	0.87 (0.12)	
Clinically active IBD	0.77 (0.15)	*P* < 0.001
Clinically inactive IBD	0.88 (0.10)	
Clinically significant insomnia	0.71 (0.16)	*P* < 0.001
No significant insomnia	0.89 (0.11)	

Comparison between merged cells by Student's *t*‐test.

### EQ‐5D component scores

Sleep quality (PSQI) and insomnia severity scores both moderately correlated with utility score, EQVAS, “usual activities,” “pain,” and “depression‐anxiety” scores, and weakly correlated with “mobility” scores (Table 3). Neither score correlated with “self‐care.” This was further investigated with PSQI scores different between most scores in components “depression‐anxiety,” “pain,” and “usual activities” (see Figure S5) and little difference in scores in other EQ‐5D components. Insomnia severity (see Supplementary Figure S6) had similar associations, although less difference seen in ISI scores across the scores for EQ‐5D component “usual activities.”

**Table 3:** EQ‐5D scores and Pearson's correlation with sleep quality (Pittsburgh sleep quality index [PSQI]), inflammatory bowel disease activity as the Harvey–Bradshaw index (HBI) for Crohn's disease, and the Simple Clinical Colitis Activity Index (SCCAI) for ulcerative or indeterminate colitis, and insomnia severity via the insomnia severity index (ISI).

EQ‐5D scores	PSQI	HBI	SCCAI	ISI
Utility score	−0.46*	−0.50*	−0.49*	−0.47*
EQVAS	−0.38*	−0.50*	0.51*	−0.43*
Mobility	0.22*	0.25*	0.24*	0.15*
Self‐care	0.075	0.09	0.097	0.039
Usual activities	0.32*	0.37*	0.36*	0.31*
Pain	0.35*	0.42*	0.40*	0.31*
Depression‐anxiety	0.37*	0.34*	0.33*	0.41*

* *P* < 0.001.

IBD activity measures by HBI or SCCAI had a strong correlation with EQ‐5D utility score and EQVAS, moderate correlation with “usual activities,” “pain,” and “depression‐anxiety” scores, and weak correlation with “mobility” scores. The general well‐being component of SCCAI and HBI had the strongest correlation with EQVAS and the EQ‐5D utility score, followed by the abdominal pain subscore of the HBI (see Table S4). Abdominal pain was more common in those with active disease in both Crohn's disease and ulcerative colitis (*P* < 0.0001), with differences seen between IBD subtypes in active disease (*P* = 0.014) but not inactive disease (*P* = 0.21) (see Table S6). Extraintestinal manifestations were also considered with all manifestations associated with a decrease in the utility score, with the largest decrease seen in oral involvement (see Table S5).

### Components of sleep quality

The EQ‐5D utility score was worse with worse PSQI components sleep quality, daytime dysfunction, sleep disturbance, and sleep duration (see Figure S7). Sleep latency, sleep efficiency, and sleep medications did not reach significance for worse “utility” scores (Figure S7). Similarly, sleep efficiency, latency, and medications did not reach above weak correlation with any EQ‐5D component (see Table S2). Further analysis of sleep quality, daytime dysfunction, sleep disturbance, and sleep duration was undertaken.

### Sleep duration

Sleep duration remained associated with domain scores “depression‐anxiety,” “pain,” and “usual activities” following adjustment by demographic variables (see Table 4). Following adjustment for IBD activity, the association with sleep duration and domain scores was attenuated, but remained for domains “pain” (*β* 0.22 [0.15–0.30]) and “depression‐anxiety” (*β* 0.15 [0.06–0.25]). After inclusion of anxiety and depression, only the “pain” domain, although further attenuated, remained significant (*β* 0.17 [0.10–0.25]).

**Table 4:** Pittsburgh Sleep Quality Index (PSQI) components and EQ‐5D domains with univariate regression and multivariate regression for each PSQI component separately.

	Univariate	Multivariate^†^	Multivariate^†^ with IBD activity	Multivariate^†^ with IBD activity, depression and anxiety
	Coefficients (95% CI)	Coefficients (95% CI)	Coefficients (95% CI)	Coefficients (95% CI)
PSQI‐sleep quality				
Depression‐anxiety	0.44 (0.32–0.55)*	0.42 (0.31–0.53)*	0.38 (0.26–0.49)*	−0.03 (−0.12 to 0.06)
Pain	0.44 (0.34–0.54)*	0.36 (0.27–0.46)*	0.29 (0.19–0.38)*	0.18 (0.09–0.29)*
Activities	0.29 (0.19–0.38)*	0.23 (0.14–0.33)*	0.19 (0.09–0.28)*	−0.012 (−0.11 to 0.084)
PSQI‐daytime dysfunction				
Depression‐anxiety	0.57 (0.45–0.68)*	0.54 (0.42–0.65)*	0.50 (0.38–0.62)*	−0.04 (−0.15 to 0.06)*
Pain	0.43 (0.32–0.54)*	0.34 (0.24–0.44)*	0.24 (0.15–0.34)*	0.09 (−0.027 to 0.20)
Activities	0.46 (0.37–0.55)*	0.40 (0.31–0.49)*	0.36 (0.27–0.46)*	0.12 (0.006–0.22)**
PSQI‐disturbance				
Depression‐anxiety	0.55 (0.41–0.69)*	0.52 (0.38–0.66)*	0.46 (0.32–0.61)*	0.0073 (−0.11 to 0.12)
Pain	0.60 (0.48–0.72)*	0.47 (0.35–0.59)*	0.36 (0.24–0.48)*	0.25 (0.12–0.37)*
Activities	0.40 (0.29–0.52)*	0.32 (0.21–0.44)*	0.26 (0.14–0.38)*	0.068 (−0.050 to 0.19)
PSQI‐duration				
Depression‐anxiety	0.20 (0.11–0.30)*	0.18 (0.09–0.29)*	0.15 (0.06–0.25)*	−0.048 (−0.12 to 0.02)
Pain	0.32 (0.24–0.41)*	0.27 (0.20–0.36)*	0.22 (0.15–0.30)*	0.17 (0.10–0.25)*
Activities	0.15 (0.07–0.23)*	0.11 (0.03–0.19)*	0.08 (0.001–0.15)**	0.015 (−0.059 to 0.088)

* *P* < 0.0001;

** *P* < 0.05.

^†^ Age, gender, IBD subtype, weight, biologic usage, smoker, alcohol, IBD surgery, opioid usage.

Multivariate regression was then conducted with demographic variables, and then sequentially with IBD activity (as a binary variable with active IBD defined by Harvey–Bradshaw Index ≥5, Simple Clinical Colitis Activity Index over 5) as well and then finally including depression and anxiety scores (Patient Health Questionnaire‐9, and Generalized Anxiety Disorder‐7).

CI, confidence interval; IBD, inflammatory bowel disease.

### Sleep disturbance

Sleep disturbance remained associated with domain scores “depression‐anxiety,” “pain,” and “usual activities” following adjustment by demographic variables. Following adjustment by IBD activity, the association with sleep duration and domain scores was attenuated, but remained relevant for all domains. After inclusion of anxiety and depression, only the “pain” domain, although further attenuated, remained significant (*β* 0.25 [0.12–0.37]).

### Daytime dysfunction

Daytime dysfunction remained associated with domain scores “depression‐anxiety,” “pain,” and “usual activities” following adjustment by demographic variables (see Table S4). Following adjustment for IBD activity, the association with sleep duration and domain scores was attenuated, but remained relevant for all domains. After inclusion of anxiety and depression, the “usual activities” domain remained significant but with a negligible coefficient (*β* 0.12 [0.0.006–0.22]).

### Sleep quality

Sleep quality remained associated with domain scores “depression‐anxiety", “pain,” and “usual activities” following adjustment for demographic variables (see Table 4). Following adjustment for IBD activity, the association with sleep duration and domain scores was attenuated, but remained relevant for all domains. After inclusion of anxiety and depression, only the “pain” domain, although further attenuated, remained significant (*β* 0.18 [0.09–0.29]).

### Insomnia

Clinically significant insomnia was present in 61.7% of the cohort, with at least moderate insomnia in 36.3%. Health‐related QoL scores (utility score and EQVAS) were significantly worse in those with clinically significant insomnia and active IBD than with active IBD alone (see Table 5). This effect may be partially explained by the presence of higher IBD activity scores (SCCAI or HBI) in those with clinically significant insomnia and active IBD than active IBD alone (see Table 5). Health‐related QoL scores were similar between those with active IBD without insomnia and those with insomnia without active IBD (see Table 5). There was no difference in the rate of clinically significant insomnia between ulcerative colitis and Crohn's disease (see Table S3).

**Table 5:** Differences in EQ‐5D utility score and EQVAS for groups active inflammatory bowel disease (IBD), active IBD with clinically significant insomnia (Insomnia Severity Index over 15), and inactive IBD.

	EQVAS [mean (SD)]	Utility score [mean (SD)]	IBD activity scores [mean (SD)]
Inactive IBD	76.37 (14.64)	0.89 (0.095)	SCCAI 4.43 (1.43) HBI 3.91 (1.06)
Clinically significant insomnia without active IBD	68.72 (16.64)	0.83 (0.15)	SCCAI 4.83 (1.34) HBI 4.50 (0.78)
Active IBD without clinically significant insomnia	66.25 (19.31)	0.82 (0.11)	SCCAI 7.24 (2.47) HBI 7.20 (2.60)
Active IBD with clinically significant insomnia	54.67 (18.87)	0.70 (0.16)	SCCAI 8.98 (2.65) HBI 9.17 (2.95)

Differences also assessed for IBD activity scores HBI (Harvey–Bradshaw Index) for Crohn's disease and SCCAI (Simple Clinical Colitis Activity Index) for ulcerative or indeterminate colitis. One‐way anova for EQVAS (df = 3, *F* = 36.97, *P* < 0.0001), all comparisons significant (*P* < 0.001), except 1v0 (*P* = 0.21) and 2v1 (*P* = 0.94). One‐way anova for utility score (df = 3, *F* = 76.64, *P* < 0.0001), all comparisons significant (*P* < 0.001), except 1v0 (*P* = 0.21) and 2v1 (*P* = 0.99). One‐way anova for SCCAI (df = 3, *F* = 138.08, *P* < 0.0001), all comparisons significant (*P* < 0.001), except 1v0 (*P* = 0.83). One‐way anova for HBI (df = 3, *F* = 159.34, *P* < 0.0001), all comparisons significant (*P* < 0.001), except 1v0 (*P* = 0.70).

SD, standard deviation.

A change in insomnia severity score of 1 was associated with a reduction of 1.4 (1–17‐1.6) (univariate regression, see Table 6) in the EQVAS, with clinically significant insomnia associated with a reduction of 13.6 (10.42–16.91) (univariate regression see Table 6) in EQVAS. Following the introduction of demographic and IBD activity, the reduction in EQVAS for clinically significant insomnia remained significant (10.11 [6.96–13.27]). However, after introduction of depression and anxiety scores, there was no significant reduction in EQVAS. Similar results were obtained when considering the influence of insomnia on the utility score (Table 6).

**Table 6:** Insomnia Severity Index score (ISI) and EQVAS with univariate regression, and multivariate regression including demographic factors, followed by inclusion of inflammatory bowel disease (IBD) activity (as a binary variable with active IBD defined by Harvey–Bradshaw Index ≥5, Simple Clinical Colitis Activity Index over 5) as well and then finally including depression and anxiety scores (Patient Health Questionnaire‐9, and Generalized Anxiety Disorder‐7).

	Univariate	Multivariate^†^	Multivariate^†^ with IBD activity	Multivariate^†^ with IBD activity, depression and anxiety
	Coefficients (95% CI)	Coefficients (95% CI)	Coefficients (95% CI)	Coefficients (95% CI)
EQVAS	−1.42 (−1.66 to −1.17)*	−1.27 (−1.52 to −1.03)*	−1.09 (−1.34 to −0.84)*	−0.24 (−0.053 to 0.040)**
EQ‐5D utility score	−0.011 (−0.013 to −0.0098)*	−0.010 (−0.012 to −0.0085)*	−0.0092 (−0.011 to −0.0073)*	−0.014 (−0.0033 to 0.00053)**

* *P* < 0.0001;

** *P* > 0.05.

^†^ Age, gender, IBD subtype, weight, biologic usage, smoker, alcohol, IBD surgery, opioid usage.

ISI and EQ‐5D utility score with univariate linear regression and multivariate regression including demographic factors, followed by inclusion of IBD activity (as a binary variable with active IBD defined by Harvey–Bradshaw Index ≥5, Simple Clinical Colitis Activity Index over 5) as well and then finally including depression and anxiety scores (Patient Health Questionnaire‐9, and Generalized Anxiety Disorder‐7).

CI, confidence interval.

## Discussion

This represents the largest study reporting on sleep quality and insomnia and its relationship with QoL in people with IBD. This study presents a detailed analysis of the relationship between health‐related QoL and sleep quality, taking into account both IBD activity and co‐existing mental health conditions. Of note, sleep quality remained associated with the QoL pain domain after adjusting for IBD activity and depression and anxiety. Also of significance, insomnia in the absence of active IBD was associated with a reduction in QoL, and this reduction was similar to magnitude to that seen with active IBD in the absence of insomnia.

Sleep quality, sleep duration, and disturbed sleep were associated with a poorer QoL, and the presence of insomnia was associated with worse QoL independent of IBD activity. Aspects of disrupted sleep such as sleep disturbance, daytime dysfunction, and sleep quality were associated with worse QoL “pain” scores following adjustment for demographic factors, IBD activity, and depression and anxiety. Similarly, sleep duration was associated with worse QoL “usual activities” score.

Pain is commonly reported by people with IBD^45^ and has been related to IBD symptoms. Chronic pain^46^ that is more complex and influenced by many factors such as psychological factors^47^ and maladaptive processes such as central sensitization are also recognized in patients with IBD.^48^, ^49^ Sleep quality has been associated with alterations in the perception of pain^50^ that perhaps explains the association with the pain domain scores seen in this study. The relationship between the impact of pain on social and cognitive function and sleep has been explored in IBD, with a conceptual model proposing important indirect effects from insomnia along with IBD activity, anxiety, and depression.^51^

Although these data suggest differences in QoL due to insomnia irrespective of IBD activity, it was not possible to establish a similar effect in relation to anxiety and depression. Insomnia is known to have a complicated and likely bidirectional relationship with anxiety and depression.^24^, ^52^, ^53^ Depression and anxiety are prevalent in people with IBD,^54^ and in this study, most of those with severe depression scores (79%) and two thirds of those with severe anxiety scores had clinically significant insomnia. A larger cohort with greater differentiation between those with insomnia and anxiety or depression may allow differences in QoL to be established.

Sleep represents a modifiable risk factor for impaired QoL. Treatment for insomnia, the most common sleep disorder in IBD,^55^ is readily available in the form for cognitive behavioral therapy in insomnia (CBTi).^56^, ^57^ A pilot study of CBTi in an IBD population has demonstrated feasibility and suggested efficacy,^25^ although larger studies are required to determine this. Other causes of sleep disturbance such as obstructive sleep apnea, noted to be more common in people with IBD than the general population,^58^ also have readily available treatment in the form of continuous positive airway pressure and other devices.^59^ Screening for sleep disturbance in IBD clinic could be considered and may be possible with typical IBD clinic data.^60^

The limitations of this study include selection bias as a result of the use of an online questionnaire and the predominantly female participants. Reporting bias is also relevant, noting a population of people with Crohn's disease self‐reported worse sleep quality than that measured objectively.^19^ The lack of an objective measure of sleep quality and IBD activity, such as endoscopic activity, is considered a limitation, as is the absence of a validated pain questionnaire.

Despite these limitations, this study contributes to the IBD QoL literature, highlighting the importance of sleep in this population. Further work should consider longitudinal data on the progression of QoL and its relationship to sleep, in particular, its prognostic value in IBD outcomes and QoL. Consideration should also be given to implementing screening for sleep disorders in IBD clinic and intervention studies directed at sleep with a view to improving QoL.

In conclusion, health‐related QoL in IBD is influenced by aspects of sleep quality irrespective of IBD activity and mental health conditions. The presence of insomnia is associated with a significant reduction in health‐related QoL. Consideration should be given to sleep targeting interventional studies in an IBD population.

## Supporting information


**Appendix S1.** Supporting information.

**Appendix S2.** Supporting information.

## Data Availability

The data underlying this article are available upon request to the author.
