# Supplementary material for: Sleep quality is associated with reduced quality of life in inflammatory bowel disease through its interaction with pain
Source: JGH Open. 2024 Aug 24;8(8):e70021. doi: 10.1002/jgh3.70021 (PMC11344164; doi:10.1002/jgh3.70021)
Supplement: Supplementary file 1 — Appendix S1. Supporting information. [file JGH3-8-e70021-s002.docx]

Histograms

Supplementary figure 1: Histogram of EQ-mobility score

Supplementary figure 2: Histogram of EQ-anxiety-depression score

Supplementary figure 3: Histogram of EQ-pain score

Supplementary figure 4: Histogram of EQ-activities score

Supplementary table 1: Inflammatory bowel disease subtypes Crohn’s disease and ulcerative colitis and Q-5D utility score, EVQAS and EQ-5D component mean scores (student t-test).

| EQ-5D scores | Crohn’s disease | Ulcerative colitis | P value |
| --- | --- | --- | --- |
| Utility score | 0.79 | 0.80 | 0.35 |
| EQVAS | 64.09 | 65.10 | 0.56 |
| Mobility | 1.51 (0.82) | 1.38 (0.75) | 0.059 |
| Selfcare | 1.13 (0.46) | 1.08 (0.34) | 0.12 |
| Usual Activity | 1.81 (0.86) | 1.71 (0.86) | 0.20 |
| Pain | 2.27 (0. 19) | 2.18 (0.96) | 0.29 |
| Depression and anxiety | 2.27 (1.08) | 2.27 (1.02) | 0.96 |

.

Supplementary figure 5: Pittsburgh sleep quality index score (PSQI) (mean, standard deviation) for EQ-5D components scores. EQ-anxiety depression ANOVA (df=4, F=24.01, p<0.0001) on post hoc Tukey test all significant (p<0.001) except 3v2, 4v3, 5v4. EQ-pain ANOVA (df=4, F=33.82, p<0.0001) on post hoc Tukey test all significant (<0.001) except 4v3, 5v3, 5v4. EQ-usual activities ANOVA (df=4, F=17.28, p<0.0001) on post hoc Tukey test significant results (p<0.04) 2v1, 3v1, 4v1, 2v2 , otherwise no differences seen. EQ-self care ANOVA (df=4, F=2.37,p=0.0519) – no significant differences between groups. EQ-mobility ANOVA (df=4, F=8.19,p<0.0001) on post hoc Tukey test significant result (p<0.03) 2v1, 4v1, other comparisons not significantly different.

Supplementary figure 6: Insomnia Severity Index (ISI) (mean, standard deviation) for EQ-5D components scores. EQ-anxiety depression ANOVA (df 4, F=30.99, p<0.0001) on post hoc Tukey test all significant except (p<0.004) except 5v4. EQ-pain ANOVA (df=4, F=26.88, p<0.0001) on post hoc Tukey test all significant (p<0.0001) except 5v2, 4v3, 5v3, 5v4. EQ-usual activities ANOVA (df=4, F=22.39, p<0.0001) on post hoc Tukey test significant result (p<0.006) 2v1, 3v1, 4v1 3v2, otherwise no differences seen. EQ-self care ANOVA (df=4, F=1.48, p=0.21) – no significant differences between groups. EQ-mobility ANOVA (df=4, F=6.09, p<0.001) on post hoc Tukey test significant result 2v1 (p=0.01) otherwise no significant differences

Supplementary Figure 7: EQ-5D utility scores for each Pittsburgh Sleep Quality Index (PSQI) component. PSQI-medications ANOVA (df=3, F=14.63, p < 0.001) on post hoc Tukey test significant results 3v0 p<0.001, 2v0 p=0.009, other comparisons not significant. PSQI-sleep quality ANOVA (df=3, F=30.94, p<0.001) on post hoc Tukey test all significant all (p< 0.002) except 1v0 (p=0.65). PSQI-sleep efficiency ANOVA (df=3, F=10.29, p<0.001) on post hoc Tukey test significant result 3v0 p<0.001, 2v0 p=0.003, otherwise not significant. PSQI-daytime dysfunction ANOVA (df=3, F=42.2, p<0.0001) on post hoc Tukey test all significant (p < 0.0001) except 1v0, 2v0. PSQI-latency ANOVA (df=3, F=9.4, p<0.0001) on post hoc Tukey test significant results: p<0.0001, 3v0 p<0.001, 2v0 p=0.020, 3v1 p=0.002, otherwise not significant. PSQI-disturbance ANOVA (df=3, F=38.05, p<0.0001) on post hoc Tukey test all significant (p< 0.003), except 1v0 not significant. PSQI-duration ANOVA (df=3, F=17.2, p<0.0001) on post hoc Tukey test all significant (p<0.0014), except 1v0 not significant.

| PSQI components | Mobility | Selfcare | Activity | Pain | Anxiety and depression |
| --- | --- | --- | --- | --- | --- |
| Duration | 0.15* | 0.018 | 0.16* | 0.27* | 0.17* |
| Disturbance | 0.23* | 0.068 | 0.27* | 0.32* | 0.31* |
| Latency | 0.084 | 0.030 | 0.17* | 0.12 | 0.19* |
| Daytime dysfunction | 0.14* | 0.080 | 0.36* | 0.25* | 0.36* |
| Efficiency | 0.16* | 0.047 | 0.18* | 0.21* | 0.13 |
| Quality | 0.10 | -0.0019 | 0.23* | 0.27* | 0.31* |
| Medications | 015* | 0.094 | 0.13 | 0.18* | 0.24* |

Supplementary table 2: EQ-5D domains and Pearson’s correlation with Pittsburgh Sleep quality index (PSQI) component scores. * p < 0.001

Supplementary table 3: Inflammatory bowel disease (IBD) subtypes and insomnia, depression, anxiety and disease activity. No significant differences were seen between IBD subtypes. Clinically active IBD was present in 67.1% of those with Crohn’s disease and 67.9% of those with ulcerative colitis or indeterminate colitis. Differences in insomnia, depression and anxiety were then considered within each IBD subtype by clinical IBD activity. ** p<0.001. * p<0.05

|  | Crohn’s disease | Active IBD | Inactive IBD | Ulcerative colitis or indeterminate colitis | Active IBD | Inactive IBD |
| --- | --- | --- | --- | --- | --- | --- |
| Clinically significant insomnia (%) | 36.6 | 47.1 | 15.0** | 38.3 | 42.6 | 13.1** |
| Clinically significant depression (%) | 20.1 | 26.8 | 6.2** | 18.9 | 25.5 | 4.8** |
| Clinically significant anxiety (%) | 31.1 | 35.6 | 22.1* | 30.7 | 38.0 | 15.5** |

Supplementary table 4: Correlation between inflammatory bowel disease symptoms subscores and EQ-5D subscores. General well-being as per Harvey Bradshaw Index and Simple clinical colitis activity index. Abdominal pain and number of liquid or soft stools scored as per the Harvey Bradshaw index. Urgency, nocturnal bowel motions and blood in stool as per the Simple clinical colitis activity index. *p < 0.05.

|  | Mobility | Selfcare | Activity | Pain | Anxiety and depression | EQVAS | Utility score |
| --- | --- | --- | --- | --- | --- | --- | --- |
| General well being | 0.29* | 0.14* | 0.48* | 0.44* | 0.36* | -0.56* | -0.52* |
| Abdominal pain | 0.19* | 0.029 | 0.27* | 0.42* | 0.25* | -0.33* | -0.38* |
| Number of liquid or soft stools | 0.035 | 0.020 | 0.11* | 0.16* | 0.12* | -0.18* | -0.17* |
| Urgency | 0.094* | 0.032 | 0.14* | 0.17* | 0.12* | -0.21* | -0.17* |
| Blood in stool | 0.045 | 0.047 | 0.13* | 0.19* | 0.097* | -0.17* | -0.18* |
| Nocturnal bowel motions | -0.017 | -0.076 | 0.053 | 0.055 | 0.084* | -0.10* | -0.093* |

Supplementary table 5: Extraintestinal manifestations of inflammatory bowel disease and EQ-5D sub-scores. Means and standard deviation reported of EQ-5D sub-scores for the presence of each extraintestinal manifestations (t-test performed). *** p<0.0001, ** p<0.001, * p<0.05

|  | Mobility | Selfcare | Usual activities | Pain | Anxiety and depression | EQVAS | Utility score |
| --- | --- | --- | --- | --- | --- | --- | --- |
| Population mean (SD) | 1.5 (0.8) | 1.1 (0.4) | 1.7 (0.8) | 2.2 (0.9) | 2.3 (1.0) | 64.5 (19.9) | 0.79 (0.15) |
| Perianal disease | 1.5 (0.8) | 1.1 (0.3) | 1.9 (0.9)* | 2.5 (0.9)** | 2.4 (1.1) | 62.2 (20.1) | 0.76 (0.16)* |
| Skin manifestation | 1.6 (0.8) | 1.1 (0.3) | 1.9 (0.9) | 2.4 (0.9)* | 2.6 (1.0)*** | 59.7 (20.3)* | 0.75 (0.16)* |
| Oral involvement | 1.5 (0.7) | 1.1 (0.4) | 2.0 (0.9)** | 2.5 (0.9)** | 2.6 (1.2)*** | 57.8 (19.0)*** | 0.74 (0.16)*** |
| Uveitis or other eye involvement | 1.6 (0.8)** | 1.1 (0.5)* | 1.9 (0.9)** | 2.4 (0.9)** | 2.4 (1.1)*** | 60.8 (20.4) *** | 0.76 (0.16)*** |
| Active arthropathy | 1.7 (0.9)** | 1.1 (0.5)* | 1.9 (0.9)** | 2.5 (0.9)** | 2.4 (1.1)*** | 60.0 (20.0) *** | 0.75 (0.11)*** |

Supplementary table 6: Abdominal pain sub-score (mean (SD)) reported by Simple clinical colitis active index and Harvey Bradshaw Index for ulcerative colitis or indeterminate colitis and Crohn’s disease respectively. ** p<0.001, *p<0.05

|  | Abdominal pain sub-score | Active IBD | Inactive IBD | Comparison across active and inactive IBD |
| --- | --- | --- | --- | --- |
| Crohn’s disease | 1.75 (0.86) | 2.00 (0.89) | 1.23 (0.48) | p< 0.0001 |
| Ulcerative colitis or indeterminate colitis | 1.73 (0.80) | 1.80 (0.80) | 1.27 (0.52) | p < 0.0001 |
| Comparison between Crohn’s disease and ulcerative colitis | p=0.54 | p=0.014 | p=0.21 |  |
