# Supplementary material for: Sleep quality is associated with reduced quality of life in inflammatory bowel disease through its interaction with pain
Source: JGH Open. 2024 Aug 24;8(8):e70021. doi: 10.1002/jgh3.70021 (PMC11344164; doi:10.1002/jgh3.70021)

Participant information

As a participant in this study, please answer the questions below. Thank you!
If you wish to participate anonymously and not the answer the questions below you may.

1) Please enter your email address: ______________________

2) Please enter your age: ______________________

3) Gender
   ◯ Female
   ◯ Male

4) Height (cm) ______________________

5) Weight (kilograms) ______________________

IBD baseline information

1) What type of inflammatory bowel disease do you have?

- ☐ Ulcerative colitis
- ☐ Crohn's disease
- ☐ Indeterminate colitis

2) Please tick any medications you are currently on for your inflammatory bowel disease?

- ☐ Mesalazine
- ☐ Sulfasalazine
- ☐ Azathioprine
- ☐ Mercaptopurine
- ☐ Methotrexate
- ☐ Tacrolimus
- ☐ Cyclosporine
- ☐ Adalimumab (Humira)
- ☐ Infliximab (Remicade)
- ☐ Ustekininumab (Stelara)
- ☐ Vedolizumab (Entyvio)
- ☐ Golimumab (Simponi)
- ☐ Tofacitinib (Xeljanz)
- ☐ Budesonide (Cortiment, Entocort, Budenofalk)
- ☐ Vitamin D
- ☐ Batrim
- ☐ Prednisolone

3) How many years have you had inflammatory bowel disease (Crohn's disease or ulcerative colitis) for?

____

4) Have you had surgery for your IBD?

- ◯ Yes
- ◯ No

5) Please tell us if you are on any of the medications below:

- ☐ Benzodiazepines such as diazepam, temazepam
- ☐ Medication for depression or anxiety such as sertraline or mirtazapine
- ☐ Melatonin
- ☐ Medication for pain such as codeine, tramadol, endone or oxycontin

6) Please list any other medications you are on:

____

7) Do you consume alcohol regularly?

- ◯ Yes
- ◯ No

8) Do you smoke cigarettes?

- ◯ Yes
- ◯ No

9) Please tells us any other medical problems you have:

____

10) Do you take anything to help you sleep? If so - please tells us what.

____

11) Do you perform shift work overnight?

- ◯ Yes
- ◯ No

IBD Disease Activity

Please answer the following questions according to the symptoms you have had over the past three days.

1) How many bowel actions have you been passing during the day time i.e. from waking up until going to bed to sleep for the last 3 days?

- ◯ 0-3 times
- ◯ 4-6 times
- ◯ 7-9 times
- ◯ >9 times

2) How many bowel actions have you been passing during night time i.e. after going to bed, for the last 3 nights?

- ◯ 0-3 times
- ◯ 4-6 times

3) For the previous day please tell us the severity of any abdominal you may have:

- ◯ None
- ◯ Mild
- ◯ Moderate
- ◯ Severe

4) What degree of urgency of defecation do you experience? i.e. when you feel the need to open your bowels, how quickly do you need to go?

- ◯ I have no urgency at all
- ◯ I have to hurry to go to the toilet
- ◯ I have to go immediately
- ◯ I have incontinence (unable to control the urge and have an accident)

5) How much blood has been in your stool?

- ◯ None
- ◯ Trace (a hint or a tiny amount)
- ◯ Moderate (occasional obvious/frank blood)
- ◯ Severe (usually obvious/frank blood)

6) How do you feel about your general health?

- ◯ Very well
- ◯ Slightly below par
- ◯ Poor
- ◯ Very poor
- ◯ Terrible

Do you have any of the following symptoms apart from your bowels?

7) Joint problems: have you had painful, red or swollen joints?
The most common joints affected are the knees, ankles or toes.

- ◯ Yes
- ◯ No

8) Eyes problems: have you had sore, red and swollen eyes?
Other symptoms include blurry vision, sensitivity to light, floaters or increased tear production?

- ◯ Yes
- ◯ No

9) Mouth problems: The most common problems are round or oval mouth ulcers which usually appear as round yellowish elevated spots surrounded by a red halo.

- ◯ Yes
- ◯ No

10) Skin problems: do you have any purple ulcers, often painful, which useful develop suddenly?
Do you have any tender, hot and red bumps which most often affect the skin on the shins, arms and legs?

- ◯ Yes
- ◯ No

11) Do you have any tears or breakdown (crack/cleft) in the skin of the anus or tender lumps (abscesses) surrounding it?

◯ Yes
◯ No

ISI - Insomina questionnaire

For each question, please select the number that best describes your answer over the past two weeks.

Please rate the current severity of any of the following sleeping problems:

		None	Mild	Moderate	Severe	Very severe
1)	Difficulty falling asleep	◯	◯	◯	◯	◯
2)	Difficulty staying asleep	◯	◯	◯	◯	◯
3)	Problems waking up too early	◯	◯	◯	◯	◯

4) How satisified or dissatisfied are you with your current sleep pattern?

◯ Very satisfied
◯ Satisfied
◯ Moderately satisfied
◯ Dissatisfied
◯ Very dissatisfied

5) How noticeable to others do you think your sleep problem is in terms of impairing the quality of your life?

◯ Not at all noticeable
◯ A little
◯ Somewhat
◯ Much
◯ Very much noticeable

6) How worried or distressed are you about your current sleep problem?

◯ Not at all worried
◯ A little
◯ Somewhat
◯ Much
◯ Very much worried

7) To what extent do you consider your sleep problem to interfere with your daily functioning (e.g. daytime fatigue, mood, ability to function at work/daily chores, concentration, memory, mood, etc.) currently?

◯ Not at all interfering
◯ A little
◯ Somewhat
◯ Much
◯ Very much interfering

PHQ-9

Over the last 2 weeks, how often have you been bothered by any of the following problems?

		Not at all	Several days	More than half the days	Nearly every day
1)	Little interest or pleasure in doing things	○	○	○	○
2)	Feeling down, depressed or hopeless	○	○	○	○
3)	Trouble falling/staying asleep, or sleeping too much	○	○	○	○
4)	Feeling tired or having little energy	○	○	○	○
5)	Poor appetite or over-eating	○	○	○	○
6)	Feeling bad about yourself or that you are a failure or have let yourself or your family down	○	○	○	○
7)	Trouble concentrating on things, such as reading the newspaper or watching television.	○	○	○	○
8)	Moving or speaking so slowly that other people could have noticed. Or the opposite; being so fidgety or restless that you have been moving around a lot more than usual.	○	○	○	○
9)	Thoughts that you would be better off dead or of hurting yourself in some way.	○	○	○	○

10) If you checked off any problem on this questionnaire so far, how difficult have these problems made it for you to do your work, take care of things at home, or get along with other people?

- ○ Not difficult at all
- ○ Somewhat difficult
- ○ Very difficult
- ○ Extremely difficult

GAD-7

Over the last 2 weeks, how often have you been bothered by the following problems?

		Not all sure	Several days	Over half the days	Nearly every day
1)	Feeling, nervous, anxious or on edge	○	○	○	○
2)	Not being able to stop or control worrying	○	○	○	○
3)	Worrying too much about different things	○	○	○	○
4)	Trouble relaxing	○	○	○	○
5)	Being so restless that it's hard to sit still	○	○	○	○
6)	Becoming easily annoyed or irritable	○	○	○	○
7)	Feeling afraid as if something awful might happen	○	○	○	○

PSQI - sleep questionnaire

The following questions relate to your usual sleep habits during the past month only. Your answers shold indicate the most accurate reply for the majority of days and nights in the past month. Please answer all questions.

1) During the past month, what time have you usually gone to bed at night (please use 24 hour time - 8pm as 2000)? ____________________

2) During the past month, how long (in minutes) has it usually taken you to fall asleep each night? ____________________

3) During the past month, what time have you usually gotten up in the morning (please use 24 hour time - 8am as 0800)? ____________________

4) During the past month, how many hours of actual sleep did you get at night? (This may be different than the number of hours you spent in bed.) ____________________

During the past month, how often have you had trouble sleeping because you...

	Not during the past month	Less than once a week	Once or twice a week	Three or more times a week
5) Cannot get to sleep within 30 minutes	◯	◯	◯	◯
6) Wake up in the middle of the night or early morning	◯	◯	◯	◯
7) Have to get up to use the bathroom	◯	◯	◯	◯
8) Cannot breathe comfortably	◯	◯	◯	◯
9) Cough or snore loudly	◯	◯	◯	◯
10) Feel too cold	◯	◯	◯	◯
11) Feel too hot	◯	◯	◯	◯
12) Have bad dreams	◯	◯	◯	◯
13) Have pain	◯	◯	◯	◯

14) Other reason please describe: ____________________

15) During the past month, how often have you taken medicine to help you sleep (prescribed or "over the counter")?
- ◯ Not during the past month
- ◯ Less than once a week
- ◯ Once or twice a week
- ◯ Three or more times a week

16) During the past month, how often have you had trouble staying awake while driving, eating meals, or engaging in social activity?
- ◯ Not during the past month
- ◯ Less than once a week
- ◯ Once or twice a week
- ◯ Three or more times a week

17) During the past month, how much of a problem has it been for you to keep up enough enthusiasm to get things done?

- ◯ No problem at all
- ◯ Only a very slight problem
- ◯ Somewhat of a problem
- ◯ A very big problem

18) During the past month, how would you rate your sleep quality overall?

- ◯ Very good
- ◯ Fairly good
- ◯ Fairly bad
- ◯ Very bad

19) Do you have a bed partner or room mate?

- ◯ Not during the past month
- ◯ Less than once a week
- ◯ Once or twice a week
- ◯ Three or more times a week

If you have a room mate or bed partner, ask him/her how often in the past month you have had:

	Not during the past month	Less than once a week	Once or twice a week	Three or more times a week
20) Loud snoring	◯	◯	◯	◯
21) Long pauses between breaths while asleep	◯	◯	◯	◯
22) Legs twitching or jerking while you sleep	◯	◯	◯	◯
23) Episodes of disorientation or confusion during sleep	◯	◯	◯	◯

24) Other restlessness while you sleep, please describe:

____

EQ-5D

Under each heading, please tick the ONE box that best describes your health today.

1) Mobility

- ◯ I have no problems in walking about
- ◯ I have slight problems in walking about
- ◯ I have moderate problems in walking about
- ◯ I have severe problems in walking about
- ◯ I am unable to walk about

2) Self-care

- ◯ I have no problems washing or dressing myself
- ◯ I have slight problems washing or dressing myself
- ◯ I have moderate problems washing or dressing myself
- ◯ I have severe problems washing or dressing myself
- ◯ I am unable to wash or dress myself

3) Usual activities (e.g. work, study, housework, family or leisure activities)

- ◯ I have no problems doing my usual activities
- ◯ I have slight problems doing my usual activities
- ◯ I have moderate problems doing my usual activities
- ◯ I have severe problems doing my usual activities
- ◯ I am unable to do my usual activities

4) Pain/discomfort

- ◯ I have no pain or discomfort
- ◯ I have slight pain or discomfort
- ◯ I have moderate pain or discomfort
- ◯ I have severe pain or discomfort
- ◯ I have extreme pain or discomfort

5) Anxiety/depression

- ◯ I am not anxious or depressed
- ◯ I am slightly anxious or depressed
- ◯ I am moderately anxious or depressed
- ◯ I am severely anxious or depressed
- ◯ I am extremely anxious or depressed

6) We would like to know how good or bad your health is TODAY.
This scale is from from 0 to 100.

100 means the best health you can imagine.
0 means the worst health you can imagine.

Please enter a number indicating how your health is TODAY.

____

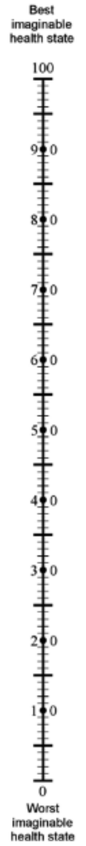

Supplement: Supplementary file 2 — Appendix S2. Supporting information. [file JGH3-8-e70021-s001.pdf]
